# Antifungal Rhizosphere Bacteria Can increase as Response to the Presence of Saprotrophic Fungi

**DOI:** 10.1371/journal.pone.0137988

**Published:** 2015-09-22

**Authors:** Wietse de Boer, Maria P. J. Hundscheid, Paulien J. A. Klein Gunnewiek, Annelies S. de Ridder-Duine, Cecile Thion, Johannes A. van Veen, Annemieke van der Wal

**Affiliations:** 1 Department of Microbial Ecology, Netherlands Institute of Ecology (NIOO-KNAW), Wageningen, 6708 PB, the Netherlands; 2 Department of Soil Quality, Wageningen University, Wageningen, 6708 PB, the Netherlands; 3 School of Biological Science, University of Aberdeen, Aberdeen, AB24 3UU, United Kingdom; 4 Insititute of Biology, Leiden University, Leiden, 2333 BE, the Netherlands; Wageningen University and Research Centre, NETHERLANDS

## Abstract

Knowledge on the factors that determine the composition of bacterial communities in the vicinity of roots (rhizosphere) is essential to understand plant-soil interactions. Plant species identity, plant growth stage and soil properties have been indicated as major determinants of rhizosphere bacterial community composition. Here we show that the presence of saprotrophic fungi can be an additional factor steering rhizosphere bacterial community composition and functioning. We studied the impact of presence of two common fungal rhizosphere inhabitants (*Mucor hiemalis* and *Trichoderma harzianum*) on the composition of cultivable bacterial communities developing in the rhizosphere of *Carex arenaria* (sand sedge) in sand microcosms. Identification and phenotypic characterization of bacterial isolates revealed clear shifts in the rhizosphere bacterial community composition by the presence of two fungal strains (*M*. *hiemalis* BHB1 and *T*. *harzianum* PvdG2), whereas another *M*. *hiemalis* strain did not show this effect. Presence of both *M*. *hiemalis* BHB1 and *T*. *harzianum* PvdG2 resulted in a significant increase of chitinolytic and (*in vitro*) antifungal bacteria. The latter was most pronounced for *M*. *hiemalis* BHB1, an isolate from *Carex* roots, which stimulated the development of the bacterial genera *Achromobacter* and *Stenotrophomonas*. *In vitro* tests showed that these genera were strongly antagonistic against *M*. *hiemalis* but also against the plant-pathogenic fungus *Rhizoctonia solani*. The most likely explanation for fungal-induced shifts in the composition of rhizosphere bacteria is that bacteria are being selected which are successful in competing with fungi for root exudates. Based on the results we propose that measures increasing saprotrophic fungi in agricultural soils should be explored as an alternative approach to enhance natural biocontrol against soil-borne plant-pathogenic fungi, namely by stimulating indigenous antifungal rhizosphere bacteria.

## Introduction

The narrow zone of soil surrounding roots, the rhizosphere, is a hotspot for activity of soil microorganisms as plant roots release a substantial amount of organic compounds, collectively named rhizodeposits, into the soil [1]. This apparent “waste” of photosynthetically obtained carbon is thought to be functionally important by stimulating the growth of microbial rhizosphere inhabitants. In this respect, much attention has been paid to so-called plant growth promoting rhizosphere bacteria (PGPR) that are metabolizing root exudates, the soluble and often most labile fraction of rhizodeposits [2]. The most mentioned benefits that plants obtain from stimulating PGPR bacteria are (1) increased availability of mineral nutrients, (2) production of plant growth stimulating compounds and (3) protection against soil-borne pathogenic fungi [3, 4].

The effects of rhizosphere bacterial communities on the functioning of the plant is strongly determined by their composition as not all bacterial species possess beneficial properties and certain bacteria do even have negative effects [2, 5]. Therefore, a major issue in rhizosphere ecology is the question if plants can steer the composition of rhizosphere bacteria in such a way that they obtain benefit from them. An argument in favor of this possibility is that plant species identity is a major factor in determining the rhizosphere bacterial composition [6]. This is attributed to plant species specific composition of root exudates selecting for certain bacterial species. However, it has also been shown that the selection by plants is strongly influenced by the bacterial community composition in the bulk soil [7]. Furthermore, abiotic soil properties and plant growth stage do influence the root exudates composition and are, therefore, also important factors in steering rhizosphere bacterial community composition [3, 1].

In addition to the aforementioned factors, other inhabitants of the rhizosphere can influence the composition of rhizosphere bacteria. Selective grazing by protozoa in the rhizosphere of *Arabidopsis thaliana* was shown to cause a drastic shift in the proportional distribution of bacterial taxa [8]. The presence of fungi can also have an impact on rhizosphere community composition. This has been most clearly shown for mycorrhizal fungi [9]. These fungi obtain their energy resources from within plant roots and produce external hyphae that explore the soil for mineral nutrients. The soil surrounding mycorrhized plant roots has been named mycorrhizosphere to distinguish it from the rhizosphere of non-mycorrhized plants [10]. For several plant species it has been shown that the composition of bacteria in the rhizosphere is different from that in the mycorrhizosphere [11, 12]. This has been mainly attributed to a change in composition of exudates when plant roots are infected by mycorrhizal fungi [13].

So far, little attention has been given to the impact of saprotrophic fungi on rhizosphere bacterial community composition. This is because root exudates are the most examined pool of rhizodeposits and bacteria have been considered to be by far the most important microbial group metabolizing these compounds [14]. However, ^13^CO_2_ pulse-labeling of plants has revealed that saptrotrophic fungi can be among the first rhizosphere inhabitants metabolizing root-derived compounds indicating that their importance as consumers of root exudates may have been underestimated [14, 15, 16]. In fact, two functional groups of saprotrophic fungi may be important root exudates consumers: real rhizosphere inhabitants (fast-growing fungi, so-called sugar fungi, that are growing on easily degradable compounds) and rhizosphere visitors (fungi specialized in the degradation of recalcitrant soil organic matter of which the exploring hyphae extend into the rhizopshere and take up exudates) [14].

We hypothesized that withdrawal of root exudates by fungi will have an impact on the competitive strategies of rhizosphere bacteria as they do not only have to deal with bacterial competitors but also with fungal competitors. In an earlier study we examined the frequency of antifungal properties of bacteria isolated from the rhizosphere of the non-mycorrhizal sedge *Carex arenaria* (sand sedge) that is occurring in both fungal-poor (bare sandy patches) and fungal-rich (understory forest) soils [17]. We observed a higher frequency of bacteria with potential antifungal properties (fungal cell wall lysing enzymes, *in vitro* antagonism) among rhizosphere bacteria isolated from fungal rich environments. However, these observations do not prove that presence of saprotrophic fungi in the rhizosphere has caused shifts in the bacterial community composition as the impact of other factors cannot be excluded.

Therefore we decided to study the possibility of selection of antifungal rhizosphere bacteria by the presence of saprotrophic fungi under controlled conditions using quartz sand microcosms, sterile *Carex* seedlings and selected fungal isolates. The results of this study, reported in this paper, do partly support our hypothesis.

## Material and Methods

### 2.1 Plants and fungi

Seeds of *Carex arenaria* (sand sedge) were collected at the edge of a driftsand location near the village Bergharen (51°10'N, 05°40'E) in the Netherlands [17]. Sterile seedlings were obtained and maintained on sterile glass beads as described by [18]. In total 5 fungal strains were used for the experiments. These were three coastal dune-soil isolates (*Fusarium culmorum* strain PvdG3, *Mucor hiemalis* strain PvdG1 and *Trichoderma harzianum* strain PvdG2) that had been used in a previous study as test fungi to determine the frequency of bacteria with antifungal properties in the *Carex* rhizosphere [17]. In addition, two other strains (*Mucor hiemalis* strain BH1B and *Trichoderma* sp. strain BH1C) were isolated from the rhizosphere/rhizoplane of *C*. *arenaria* plants that were excavated from location Bergharen. Isolation was done by cutting 1 cm young (whitish) root-pieces with adhering sand and placing them on PDA (19.5 g L^-1^ potato-dextrose agar containing a mixture of 4 antibiotics (streptomycin 100 mg L^-1^, chloramphenicol 50 mg L^-1^, oxytetracyclin 50 mg L^-1^ and ampicillin 50 mg L^-1^) to prevent bacterial growth. Incubation was at 20°C. Fungi that grew from the *Carex* root pieces as well as the fungal dune soil isolates were transferred several times to fresh antibiotic-containing PDA. Hyphal fragments were collected from antibiotic-containing PDA and were microscopically inspected for the presence of bacteria after DAPI-staining [19]. The identity of the fungi was confirmed by sequencing part of the 18S rDNA and/or ITS. Sequences are deposited in Genbank (accession numbers: KC888987–KC888990). All permissions needed for the sampling of seeds, roots and rhizopshere soil were given by Hans Hengeveld (district manager of Geldersch Landschap and Kasteelen).

For the preparation of spore suspensions fungi were grown on OA (24 g L^-1^ Oatmeal agar (Difco)) containing antibiotics (see above). Incubation was for 3 weeks at 20°C. Spores and mycelia fragments were collected by adding 10 ml sterile water and scraping the mycelial covered surface with a sterile glass rod. Spores were separated from mycelial fragments and agar fragments by filtering the suspensions through sterile glass wool. The filtered spore suspension was concentrated by centrifugation and spores were counted microscopically. Spores suspensions were stored at 4°C until use.

### 2.2 Experimental design

Petri dishes (8.5 cm diameter) were filled with 100 g autoclaved acid-washed quartz sand (granulation 0.1–0.5 mm; Honeywell Speciality Chemicals Seelze GmbH, Seelze, Germany) containing 10% (w/w) filter-sterilized (0.2 μm) nutrient-solution with the following composition (g L^-1^ water): MES (2-(*N*-morpholino)ethanesulfonic acid), 5.85; Ca(NO_3_).4H_2_O, 3.23; MgSO_4_.7H_2_O, 1.89; K_2_SO_4_, 0.73; KH_2_PO_4_, 0.25; Na_2_-EDTA, 0.013; H_3_BO_3_, 0.010; FeCl_2_.4H_2_O, 0.006; MnCl_2_.4H_2_O, 0.006; Na_2_Mo_4_.2H_2_O, 0.0005; ZnCl_2_, 0.0004; CuCl_2_.2H_2_O, 0.0002; NiCl_2_.6H_2_O, 0.0001; CoCl_2_.6H_2_O, 0.0001. Nutrient additions, with the exception of MES, were based on plant growth experiments in dune sand [20]. MES-buffer was added because acid-washed sand has no buffering capacity. Before sterilization the pH of the nutrient-solution was adjusted to 6.5 with NaOH.

Before *Carex* seedlings and bacteria were introduced fungal-rich and fungal-poor treatments were established. Five different fungal-rich treatments were created namely by introducing each of the five fungal strains described above in separate treatments. The nutrient solutions used to create fungal-rich sand did not only contain the nutrients described above (used for fungal-poor treatments) but also glucose (5 g L^-1^) and fungal spores (10^8^ L^-1^). The suspensions containing fungal spores were added after filter-sterilization of the glucose-containing nutrient solution.

Sand was mixed well with the different (with and without fungal spores) nutrient solutions and distributed well in the Petri dish bottoms. Lids were put on the Petri dish bottoms and sealed with two layers of Parafilm. Incubation was for one week at 20°C. Preliminary experiments had shown that all glucose had been metabolized by fungi at that time (based on detection of glucose with a glucose oxidase kit, Sigma). Petri-dishes were put in a vertical position in a metal tray and holes of approximately 0.3 cm dia were made on top through the overlapping zone of lid and bottom using a soldering iron.

*Carex s*eedlings were carefully and aseptically inserted through these holes in the sand. Before introduction of the *Carex* seedlings their roots had been dipped in 5 μm filtrate of a soil suspension (1: 5 in MES buffer; pH 6.5) of *Carex* rhizosphere soil collected in driftsand locations Loonse Duinen and Bergharen (see below). This filtered soil suspension served as a fungal-free inoculum of indigenous *Carex* rhizosphere bacteria. The presence of bacteria (about 10^6^ mL^-1^ bacterial colony forming units) and absence of fungi in this filtered suspension was confirmed with dilution plating on 1/10 strength TSB agar, pH 6.5 [7].

**Fig 1:**
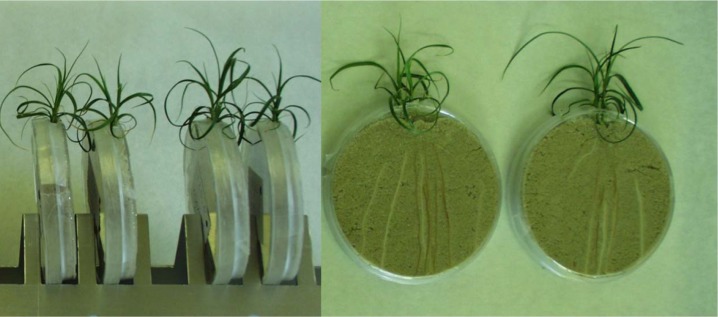
Pictures of the experimental set-up of sand microcosms with *Carex arenaria* (sand sedge) plants.

The Petri-dishes (+ plants) were covered with tin foil to prevent growth of algae and placed in a climate chamber at 19°C/ 16°C with light (8000 lux) for 16 h per 24 h and a relative humidity of 70%. Incubation was for 6 weeks. Water loss via evaporation by plants was determined by weight loss and compensated by adding aseptically sterile demineralized water in the drilled holes. A picture of the set-up is shown in Fig 1.

The experiments were performed in two runs, hereafter referred to as experiment 1 and experiment 2. Experiment 1 comprised the three coastal dune soil fungal isolates. This experiment had four treatments in total, namely: planted Petri dishes with bacteria only, planted Petri dishes with bacteria plus either one of the three fungal isolates. Experiment 2 comprised of the two indigenous (location Bergharen) fungal isolates obtained from *Carex* roots. This experiment had similar treatments as the first experiment making a total of three treatments. The filtered rhizosphere soil suspensions used as bacterial inocula were different for the two experiments: i.e. for experiment 1 the suspension was made of organic-poor sandy soil from location Loonse Duinen [7] whereas for experiment 2 organic-poor sandy soil was sampled in location Bergharen [17]. For all treatments eight replicates were prepared, but only three per treatment were used for isolation and characterization of bacteria as described below. The higher number of replicates that was prepared initially was aimed to compensate for possible dying of *Carex* seedlings. Unplanted Petri dishes with any of the five fungal isolates were harvested at the beginning of the experiments to determine the initial fungal biomass and to do another check for possible contamination with bacteria (plating of sand suspension).

### 2.3 Bacterial numbers and fungal biomass

At harvest (6 weeks) the Petri dishes were opened and the plants were removed carefully and shaken gently. Sand adhering to the roots was considered as rhizosphere. Rhizosphere sand was collected with a sterile brush. Samples were also taken from the sand that remained in the Petri dishes.

Bacterial numbers in sand samples were enumerated after suspending the sand in 0.25 g L^-1^ KH_2_PO_4_, pH 6.5 (ratio sand/buffer 1:10) and dilution plating on 1/10 strength TSB agar, pH 6.5 [7].

The development of fungal biomass was based on quantification of ergosterol, a sterol occurring in the fungal cell membrane. Ergosterol was extracted from sand using an alkaline-extraction protocol and determined by HPLC as described by [21].

### 2.4 Frequency of bacteria with potential antifungal properties

For each treatment three microcosms were randomly selected to isolate bacterial strains (40 per replicate). Bacterial isolates were randomly selected from the most abundant bacterial colonies (colonies developing from bacterial cells that were present in the most diluted sand suspensions). Bacterial isolates were individually screened for the possession of enzymes that could be involved in destabilization of fungal cell walls namely chitinases, ß-1,3- glucanases and proteases [22]. Tests for the production of chitinases and ß-1,3- glucanases were done as described by [17], except that the agar for ß-1,3- glucanase detection contained 0.5 g L^-1^ laminarin (Sigma,) instead of lichenan. Proteolytic activities of bacterial isolates was tested by the production of clear zones (haloes) on 1/10 strength TSB agar containing 80 ml L^-1^ skimmed milk.

The bacterial isolates were also screened for in vitro antagonistic activities against the fungi used in this study as well as against the plant pathogenic fungus *Rhizoctonia solani* (anastomosis group 2.2IIIB). The in vitro antagonism tests were performed on 1/10 strength TSB agar as described by De Boer et al. [17].

### 2.5 Frequency of bacterial identities

A grouping was made on basis of the morphology of the most abundant bacterial colonies, and the frequency of the different colony-types was calculated. Several (3 to 10) isolates of the most dominant bacterial colony morphologies (> 5% of total colonies) were identified by sequencing parts of the 16S rDNA gene. Primers pA (5′-AGAGTTTGATCCTGGCTCAG-3′, *Escherichia coli* positions 8 to 27) [23] and 1492R (5′-TACCTTGTTACGACTT-3′, *E*. *coli* positions 1507 to 1492) were used to amplify the nearly complete 16S rDNA gene [24]. PCR-conditions were: 94°C for 2 min, followed by 39 cycles of denaturation at 94°C for 30 s, annealing at 55°C for 60 s, and extension at 72°C for 90 s + 1 s/cycle, and ending with an extension step at 72°C for 10 min. The PCR products were analyzed on 1.5% (w/v) agarose gels, purified with QIAquick PCR purification kit (Qiagen) and sequenced as a single extension with primer 1492r by Macrogen Inc. (Korea) using an ABI 3730 XL DNA Analyzer (Applied Biosystems).

The sequences were manually edited and corrected prior to BLAST (blastn) search against the nucleotide database at NCBI (http://www.ncbi.nlm.nih.gov/blast). Identification was based on the best blastn match. Clones identified to genus level had similarities of 97% and higher. Sequences representing the major groups of bacteria found in experiment 1 and 2 are deposited in Genbank accession numbers: KC888979 –KC888986 and accession numbers: KC888967- KC888978, respectively.

### 2.6 Statistical analysis

Unless otherwise stated one-way analysis of variance (ANOVA) was used to test differences in averages of bacterial numbers and of frequencies of bacterial properties between fungal and control treatments at the 5% probability level. The assumption of normality was tested with Shapiro-Wilk statistics and homogeneity of variance was assessed with Levene’s test. In case of violation of the assumption of normality or homogeneity of variances data were Log transformed. Differences in average values of fungal biomass (ergosterol) between root-adhering) and non root-adhering sand were also tested with ANOVA.

## Results

### 3.1 Experimental conditions, fungal biomass and bacterial numbers

Not all *Carex* seedlings survived the introduction in the sand-filled Petri dishes but the aim to have at least three replicates per treatment was met. At harvest (6 weeks), the average dry weight of the *Carex* plants was 0.3 g (root/shoot ratio 1.9) with no significant differences between treatments and none of the plants showing symptoms of disease or nutrient shortage. The addition of MES-buffer (pH 6.5) in the nutrient solution had worked well as the pH of the sand (measured in 1: 2.5 suspensions in water) at harvest varied between 6.0 and 6.2.

Despite pre-culturing of fungi on agar containing antibacterials and microscopic inspection of fungal hyphae for presence of bacteria, two fungi namely *F*. *culmorum* (experiment 1) and *T*. *harzianum* (experiment 2) appeared to be contaminated with bacteria as became apparent from bacterial colony counts in sterile sand containing only these fungi as inoculants. The introduction of bacteria by fungi themselves is interfering with the aim to study possible selective pressure by fungi on the development of inoculated soil bacteria in the *Carex* rhizosphere. Therefore, treatments with these two fungi were excluded from data analysis. The remaining experiment consisted of two fungi (*T*. *harzianum* strain PvdG2 and *M*. *hiemalis* strain PvdG1) in experiment 1 and one fungus (*M*. *hiemalis* strain BH1B) in experiment 2.

**Fig 2:**
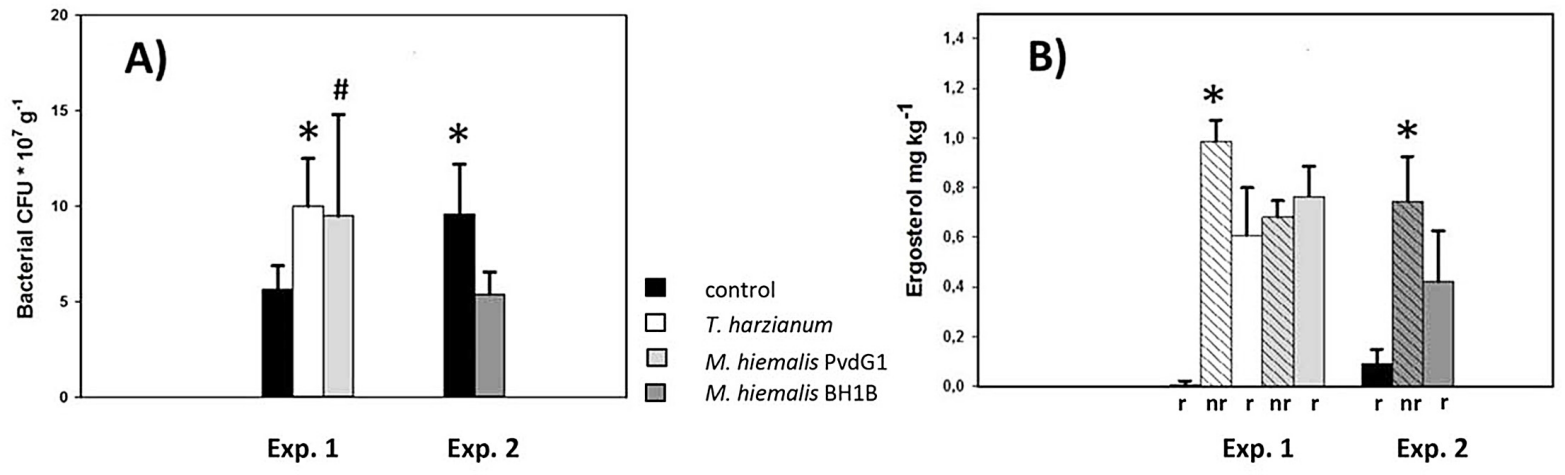
Bacterial numbers and fungal biomass (ergosterol) after 6 weeks of growth of *Carex arenaria* seedlings in quartz sand microcosms. 1A: Number of bacterial colony forming units in the *Carex* rhizosphere (root-adhering sand); * indicates significant difference (p < 0.05) between microcosms with and without (control) the presence of inoculated fungi, # indicates p = 0.052 for Log-transformed data. 1B: Ergosterol concentrations. r indicates rhizophere sand (sand adhering to *Carex* roots), nr indicates sand remaining after removal of *Carex* roots. * indicates significant difference (p < 0.05) within fungal treatments between root-adhering and non-root-adhering sand. Data for both figures are the averages of 5 or 6 sand microcosms. Error bars represent standard deviation.

At harvest, bacterial colony forming units in the rhizosphere of *Carex* plants varied from 0.5 to 1.0 *10^8^ CFU per gram dry soil. In experiment 1 the numbers of rhizosphere bacteria were higher in the presence of fungi than in the controls without fungal pre-inoculation, whereas this was the reverse in experiment 2 (Fig 2A).

The level of the fungal biomarker ergosterol remained low in the controls that had been inoculated with filtered soil suspension indicating no or negligible fungal growth originating from the *Carex* seedlings or the filtered soil suspension (Fig 2B). Pre-growth of the selected fungal strains in glucose-containing sand resulted in ergosterol concentrations that varied from 0.5 to 1.0 mg kg^-1^ (Fig 2B). The ergosterol concentrations in the rhizosphere sand of *T*. *harzianum* and *M*. *hiemalis* BH1B were lower than in the sand remaining after removal of the roots, but not for *M*. *hiemalis* PvdG1 (Fig 2B).

### 3.2 Frequency of potentially antifungal bacteria

**Fig 3:**
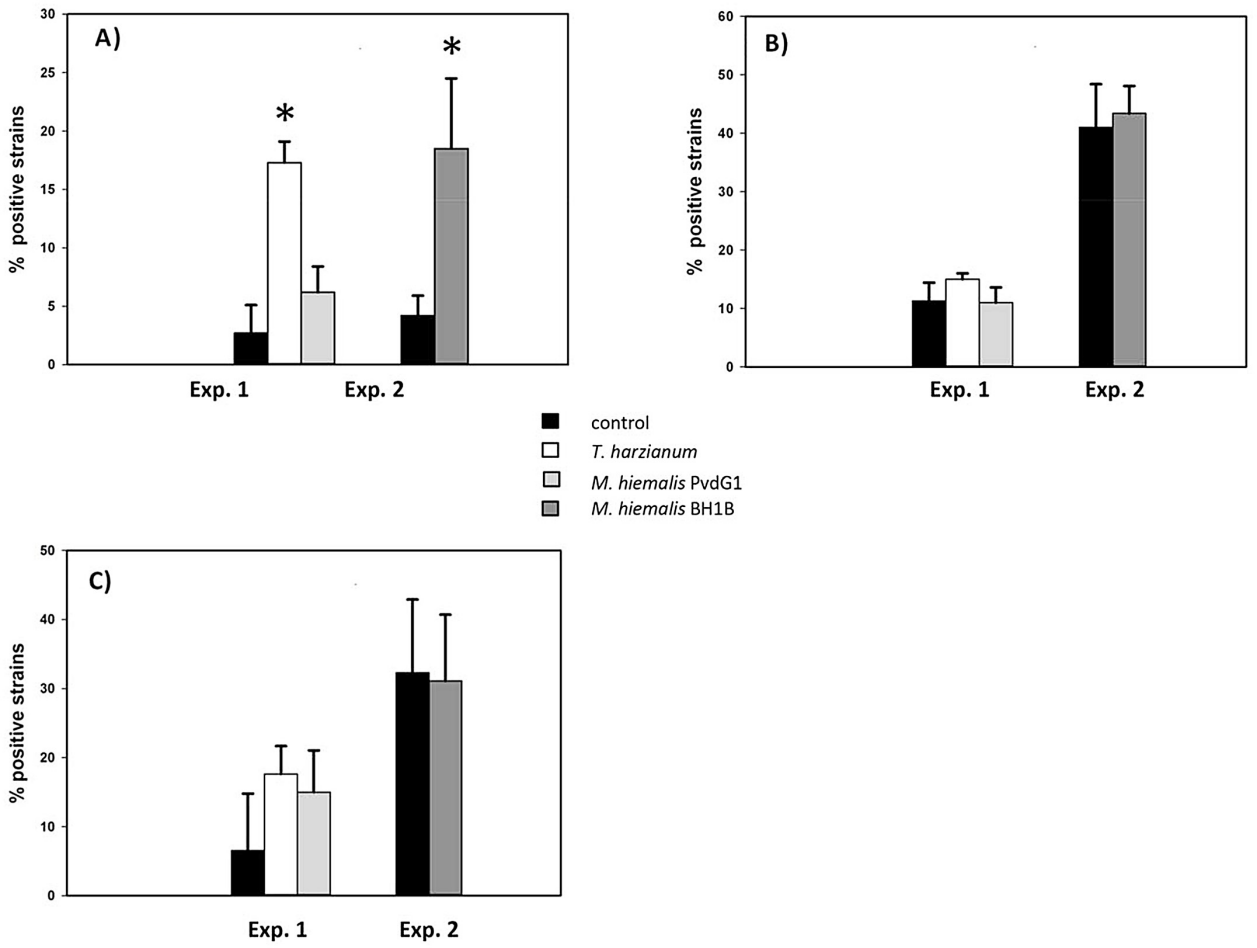
Percentage of rhizosphere bacterial isolates positive for different enzyme activities. Bacterial isolates were obtained from root-adhering soil after 6 weeks of growth of *Carex arenaria* seedlings in quartz sand microcosms. * indicates significant difference (p < 0.05) between microcosms with and without pre-inoculation of fungi. Note that experiment 1 and 2 started with different bacterial inoculums as indicated in Material & Methods. Data are the averages of three randomly selected sand microcosms. Error bars represent standard deviation. For each microcosm 40 bacterial isolates were individually screened for the different enzyme activities.

The presence of the fungi in sand resulted in an increase of rhizosphere bacterial isolates with chitinolytic abilities (Fig 3A). However, this was only significant for *T*. *harzianum* (experiment 1) and *M*. hiemalis BHB1 (experiment 2) but not for *M*. *hiemalis* PvdG1 (experiment 1).

**Fig 4:**
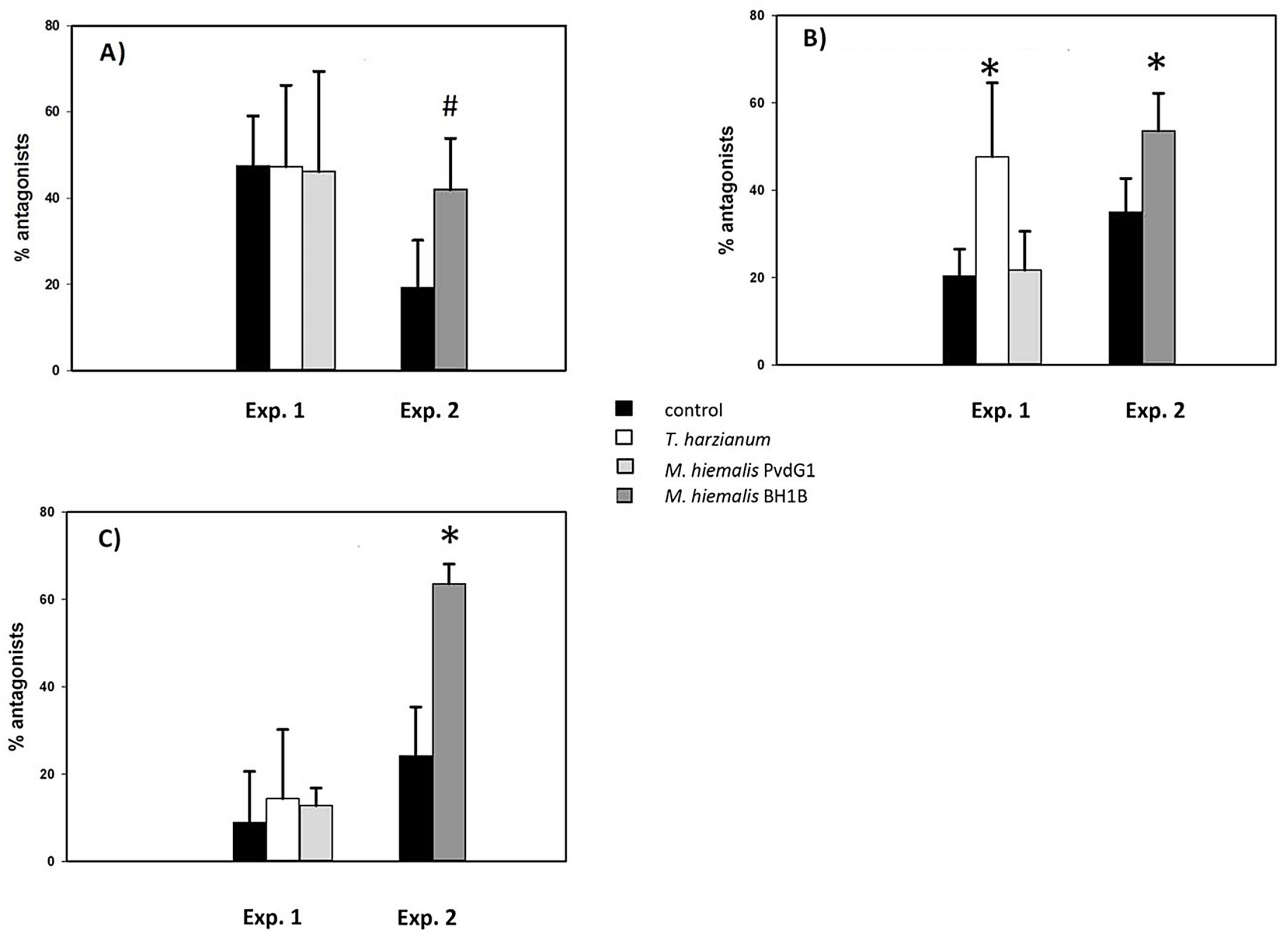
Percentage of rhizosphere bacteria isolates with *in vitro* antagonistic activity against different fungi. Bacterial isolates were obtained from root-adhering soil after 6 weeks of growth of *Carex arenaria* seedlings in quartz sand microcosms. * indicates significant difference (p < 0.05) between microcosms with and without pre-inoculation of fungi, for the ANOVA test of data of the white column in Fig 3B Log transformation was applied; # indicates p = 0.072. Note that experiment 1 and 2 started with different bacterial inoculums a as indicated in Material & Methods. Data are the averages of three randomly selected sand microcosms. Error bars represent standard deviation. For each microcosm 40 bacterial isolates were individually screened for in vitro antagonisms against the different fungi.

The frequency of bacteria possessing ß-1,3- glucanase or protease activity was not significantly affected by the presence of any of the fungi (Fig 3B and 3C). The effect of the presence of fungi in sand on the frequency of potential antifungal bacteria (*in vitro* antagonism tests) was different for each fungus (Fig 4). Presence of *M*. *hiemalis* PvdG1 (experiment 1) did not result in frequencies of *in vitro* antifungal bacteria that were significantly different from the control. Presence of *T*. *harzianum* PvdG2 (experiment 1) resulted in an increase of bacteria with antagonistic properties against itself but not against two other fungi (*M*. *hiemalis* and *R*. *solani*) tested. The presence of *M*. *hiemalis* BHB1 (experiment 2) resulted in higher frequencies of bacteria with *in vitro* antifungal activities against all three fungi tested, albeit it only significant at the 5% level for *T*. *harzianum* and *R*. *solani* (Fig 4).

### 3.3 Bacterial community composition

The presence of fungi coincided with a shift in cultivable bacterial community composition. This was most obvious for experiment 2 in which two dominant colony types (39 and 14% for colony types M2-B and M2-A, respectively) were observed that were among the minor (< 5%) colony types in the non-fungal controls (Table 1). The most dominant colony-type (M2-B) consisted of bacteria that were proteolytic and showed antagonistic activity against *M*. *hiemalis* and *R*. *solani*. Chitinolytic activity could not be detected and β-1,3-glucanase activity varied among isolates. Sequencing of several representatives of this colony-type revealed that they consisted of bacteria belonging to the genus *Achromobacter*. The other colony-type (M2-A) consisted of bacteria that were chitinolytic, proteolytic and possessed *in vitro* anti-fungal activity against all three fungi tested. Representatives of this colony-type were identified as *Stenotrophomonas* sp. Most of the *Stenotrophomonas* isolates were not able to degrade laminarin (β-1,3-glucanase activity).

**Table 1:** Identification and (potential) antifungal properties of rhizosphere bacteria representing the most dominant colony morphologies in the rhizosphere of *Carex arenaria* in sand microcosms with or without the presence of the fungi *Trichoderma harzianum* strain PvdG2 (experiment 1) or *Mucor hiemalis* strain BHB1 (experiment 2).

Treatment*	Code Colony type	Frequency of colony-type†(%)	Chitinase‡	Protease‡	Anti-fungal◊	Identification**
**Exp. 1**						
Control	C1-A	43	-	(±)	(±)	*Pedobacter*, *Pseudomonas*, *Arthrobacter*
	C1-B	12	-	-	(±)	*Mucilaginibacter*
*Trichoderma*	T1-A	37	-	(±)	(±)	*Pedobacter*, *Pseudomonas*, *Arthrobacter*
	T1-B	14	(±)	+	T (±)	*Pedobacter*,*Dyella*
**Exp. 2**						
Control	C2-A	45	-	(±)	(±)	*Pseudomonas*, *Bosea*, *Rhizobium*, *Herbaspirillum*
	C2-B	9	-	+	(±)	*Pedobacter*
*Mucor*	M2-A	14	+		M,T,R	*Stenotrophomonas*
	M2-B	39	-	+	M,R( (±)	*Achromobacter*
	M2-C	8	-	(±)	(±)	*Burkholderia*, *Bosea*,

* Treatments consisted of planted sand microcosms without (control) and with presence of fungi. Colony-types of in the treatment of *M*. *hiemalis* strain PvdG1 (exp. 1) have not been identified as the frequency of colony types was not different from the control.

^†^ Frequencies of bacterial colony-types in the *Carex*-rhizosphere at harvest (6 weeks).

^‡^ Presence or absence of the property for all strains is indicated with + and-, respectively. Variation of the property among strains is indicated with (±). Information for ß-1,3-glucanase activity is not included as for all colony types strains showed variation for this property.

^◊^ Capital letters M, T and R indicate that all strains examined had *in vitro* antagonistic activity against *Mucor hiemalis*, *Trichoderma harzianum* and *Rhizoctonia solani*, respectively. Variation among strains for *in vitro* antifungal activity is indicated with (±).

** Sequences representing the major groups of bacteria found in experiment 1 and 2 are deposited in Genbank accession numbers: KC888979 –KC888986 and accession numbers: KC888967- KC888978, respectively.

The presence of *T*. *harzianum* in experiment 1 coincided with a small increase of two types of bacterial colonies that were identified as *Pedobacte*r sp. and *Dyella* sp., respectively, and showed antagonistic activity against *T*. *harzianum* but varied in their antagonistic activity against the other fungi (Table 1). The presence of *M*. *hiemalis* PvdG1 in experiment 1 did not result in a shift of colony types as compared to the control. Therefore, no identification of bacterial colonies was done for this treatment.

## Discussion

### 4.1 Effect of saprotrophic fungi on composition of rhizosphere bacteria

Our study indicates that the presence of saprotrophic fungi in the rhizosphere can have a selective effect on the composition and functioning of bacteria. Pre-inoculation of the microcosms with *T*. *harzianum* strain PvdG2 and *M*. *hiemalis* strain BHB1 gave rise to significantly higher frequencies of rhizosphere bacterial strains possessing antifungal (*in vitro* inhibition) and potential antifungal (chitinases) properties. Fungal-induced competitive pressure on rhizosphere bacteria could explain the observed shift in bacterial community composition. In the absence of fungi, bacteria compete for root exudates with other bacteria, whereas in the presence of fungi they have to compete with both bacteria and fungi. The most successful competitive strategies for bacteria may, therefore, differ between these two situations. The increased frequency of bacteria with (potential) antifungal properties may be a reflection of the importance of these properties for bacteria during interference competition with fungi. In an earlier field study, we observed higher frequencies of potentially antifungal bacteria in the rhizosphere of *C*. *arenaria* plants growing in fungal-rich sites as compared to plants growing in nearby fungal-poor sites [17]. All together the results indicate that not only plant and soil factors but also the abundance of saprotrophic fungi may be an important factor in shaping rhizosphere bacterial communities.

Alternatively, the presence of fungi may have selected for mycophagous bacteria, i.e. bacteria growing at the expense of living fungal propagules [25]. In particular, the increase of chitinolytic bacteria could point in this direction as chitinases are involved in destabilization and degradation of chitin, an important fungal cell wall constituent. Mycophagous growth has been extensively studied for the genus *Collimonas* [26]. *Collimonas* bacteria are both chitinolytic and antifungal and do increase in sand microcosms where invading fungal hyphae are the only resources that can support growth [19]. One of the chitinolytic and antifungal bacterial genera, *Stenotrophomonas*, which strongly increased in the *Carex* rhizosphere, was also tested in that study but no fungus-induced growth was observed [19]. Therefore, selection for the best competitive strains seems to be the most likely explanation for increase of chitinolytic and antifungal bacteria in the *Carex* rhizosphere in fungus-containing microcosms. However, also under such conditions consumption of fungal material by competing bacteria is possible namely of fungal hyphae that are killed during interference competition [27].

The presence of fungi did not increase the frequency of β-1,3-glucanase producing bacteria. These enzymes have been indicated as potentially important enzymes for bacteria to attack fungi by hydrolyzing the β-1,3-glucans in the fungal cell wall [28]. In the current study the frequency of bacteria positive for β-1,3-glucanase activity did not increase in any of the fungal treatments. Moreover, antifungal *Stenotrophomas* and *Achromobacter* bacteria which had strongly increased in the fungal-enriched microcosms in experiment 2 varied with respect to β-1,3-glucanase activity. Hence, we did not obtain evidence for the importance of β-1,3-glucanases in antagonistic interactions of rhizosphere bacteria against fungi. A recent comparison of genomes of chitinolytic bacteria revealed that many bacteria from genera known for antifungal activities do not possess genes encoding β-1,3-glucanases [29]. The frequency of protease-excreting rhizosphere bacteria was also not significantly increased by the presence of fungi. However, since all isolates belonging to the antifungal genera *Stenotrophomas* and *Achromobacter* were positive in the protease assay, this can still be in support of claims that proteases can be involved in antifungal activities of bacteria [30].

In contrast to *T*. *harzianum* and *M*. *hiemalis* BHB1, the other *M*. *hiemalis* strain (PvdG1) had no significant positive effect on the frequencies of chitinolytic and antifungal bacteria. The lack of bacterial response to *M*. *hiemalis* coincided with a non-significant difference in ergosterol-based fungal biomass levels between rhizosphere sand and sand remaining after removal of roots, whereas ergosterol levels were significantly decreased in the rhizosphere sand of the two other fungi. The fact that a decrease in fungal biomass in the rhizosphere is coinciding with an increase of potentially antifungal bacteria, is another indication for antagonistic interactions between bacteria and fungi in the *Carex* rhizosphere. It is not clear why *M*. *hiemalis* PvdG1 did not trigger a similar response in *Carex* bacterial rhizosphere communities as *M*. *hiemalis* BHB1. It may be that the fungal strains differed in their possibilities to exploit *Carex* root exudates as *M*. *hiemalis* PvdG1 was originally isolated from *Ammophila* (marram grass) roots and not, as was the case for *M*. *hiemalis* BHB1, from the roots of *Carex*. To examine this, incorporation of ^13^C from labeled root exudates into fungal biomarkers should have been examined [16], but that was not included in the current experimental design.

Whereas our experiments deal with the impact of saprotrophic fungi on rhizosphere bacterial community composition, several other studies have focused on the impact of mycorrhizal fungi [31]. The bacterial community composition in the mycorrhizosphere (roots colonized by mycorrhizal fungi) is different from that in the rhizosphere indicating a selective effect of mycorrhizal fungi [11, 13, 32, 33]. This selective effect by mycorrhizal fungi on rhizosphere bacteria is likely to be different than the selective effect exerted by saprotrophs. Mycorrhizal fungi provide bacteria with nutrients (exudates) and/or can alter the quantity and quality of root exudates [9, 10]. Saprotrophs can tap from the same pool of root exudates as bacteria do [14, 16]. Hence, whereas antagonism is expected to be the predominant type of interaction between rhizosphere bacteria and saprotrophs, this is not necessarily the case for the interaction with mycorrhizal fungi. Indeed, many bacteria having a positive effect on mycorrhizal infection, so-called “helper bacteria, have been isolated from mycorrhizospheres [31]. However, as indicated by [34], the actual knowledge on trophic interactions between mycorrhizal fungi and associated bacteria is still limited.

### 4.2 Risk of the experiments

The current study involved cultivable bacteria only. The use of cultivable bacteria enables determination of frequencies of cultivable bacteria with (potential) antifungal activity. Since many known but also unknown genes can be responsible for antifungal activity it is not possible to determine these frequencies in the total bacterial DNA pool. Yet, the question remains if the results of the current study can be extrapolated to total bacterial rhizosphere communities that have developed in the sand microcosms. We think that this is the case. In an earlier study we showed that cultivable bacteria in the *Carex* rhizosphere in the natural environment form a significant part (average 35%, 10 field sites) of the total rhizosphere bacterial community [7]. In addition, we used sterile sand microcosms that were colonized by bacteria from a soil inoculum and it has been shown earlier that (re)colonization by bacteria of sterile and partially sterilized soil can result in high cultivable/total cell ratios [35, 36].

### 4.3 Perspectives for biocontrol

**Fig 5:**
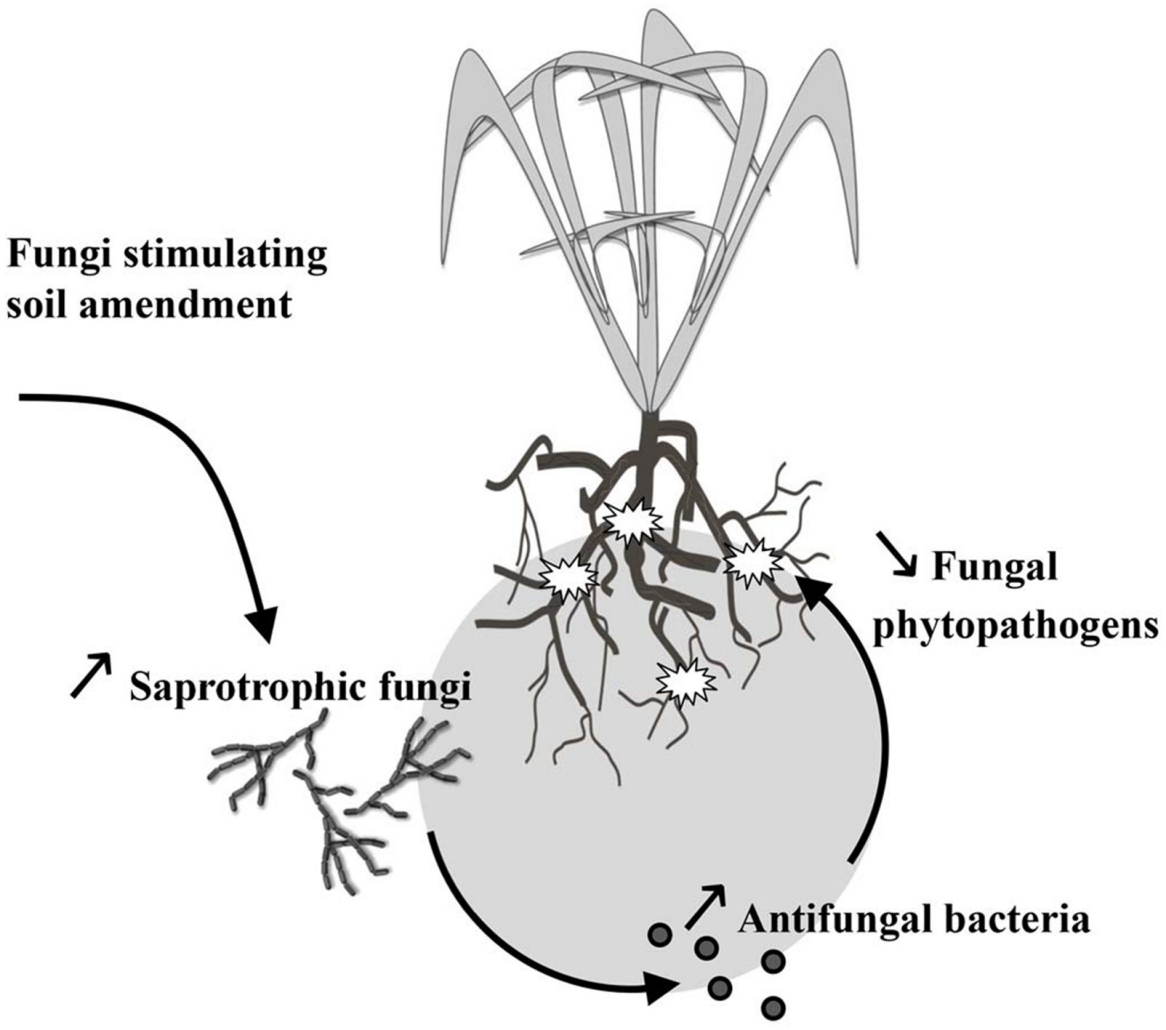
Schematic illustration of possible stimulation of biocontrol of soil-borne pathogenic fungi by increase of saprotrophic fungi. Organic amendments and/or other measures that stimulate growth of saprotrophic fungi can result in an increase of uptake of rhizodeposits by these fungi and, consequently, in an increase of competitive fungal pressure towards rhizosphere bacteria. As a result bacteria that are antagonistic against fungi will increase and several of these bacteria may also be antagonistic against soil-borne pathogenic fungi and form a natural barrier against fungal diseases. An advantage over introduction of antifungal biocontrol strains is that the fungus-induced stimulation occurs *in situ* with indigenous soil bacteria that are adapted to the local environmental conditions.

The selection of antifungal rhizosphere bacteria by saprotrophic fungi could form the basis for a novel approach to enhance natural biocontrol (Fig 5). Stimulation of indigenous bacteria with biocontrol activities has a clear advantage over the addition of biocontrol strains, namely the fact that the former are adapted to the local abiotic and biotic field situation [37]. Several studies showed that the inoculation of soil with spores or sclerotia of phytopathogens led to the emergence of antifungal bacteria [38, 39]. Obviously, introduction of propagules of phytopathogens to select indigenous antifungal bacteria is too risky to be applied as a biocontrol measure. Our study indicates that fungal-induced selection of antifungal bacteria may also be triggered by non-harmful saprotrophic fungi. In particular, the introduction of *M*. *hiemalis* BHB1 triggered antifungal bacteria that did also inhibit a pathogenic fungus. One of the bacterial genera that increased due to the presence of this *Mucor* strain is *Stenotrophomonas*. Several members of this bacterial genus are known to have biocontrol properties and are even marketed as biocontrol products [40]. Hence, it seems attractive to examine if stimulation of saprotrophic soil fungi in arable land, e.g. via organic fertilizers, will result in an increase of antifungal rhizosphere bacteria and if this results in an increased natural protection of the crop against soil-borne pathogenic fungi. The fact that we found different results with the two other fungal strains (no increase of antifungal bacteria or only of narrow-spectrum inhibiting bacteria) indicates that the fungal-induced bacterial biocontrol is by no means general. However, in real agro-ecosystems increase of saprotrophic fungi in the rhizosphere is not restricted to single species [41]. Hence, a multitude of competitive interactions between fungal and bacteria species is to be expected including the triggering of broad-spectrum inhibiting bacteria. Our previous field inventory where fungal-poor and fungal-rich *Carex* rhizospheres in natural ecosystems were compared did indeed reveal that a wide range of potential antifungal functionalities of bacteria was increased in the fungal-rich rhizospheres [17].

### 4.4 In conclusion

The presence of saprotrophic fungi in the rhizosphere can result in an increase of bacteria with antifungal properties. This observation forms a promising basis for alternative approaches to stimulate natural biocontrol.

## Supporting Information

S1 Appendix Raw data of Fig 2: Bacterial numbers and fungal biomass (ergosterol) after 6 weeks of growth of *Carex arenaria* seedlings in quartz sand microcosms. (XLS)

S2 Appendix Raw data of Figs 3 and 4: Percentage of rhizosphere bacterial isolates positive for different enzyme activities and for *in vitro* antagonistic activity against different fungi. (XLS)
